# Genes and Longevity of Lifespan

**DOI:** 10.3390/ijms23031499

**Published:** 2022-01-28

**Authors:** May Nasser Bin-Jumah, Muhammad Shahid Nadeem, Sadaf Jamal Gilani, Fahad A. Al-Abbasi, Inam Ullah, Sami I. Alzarea, Mohammed M. Ghoneim, Sultan Alshehri, Aziz Uddin, Bibi Nazia Murtaza, Imran Kazmi

**Affiliations:** 1 Biology Department, College of Science, Princess Nourah Bint Abdulrahman University, Riyadh 11671, Saudi Arabia; mnbinjumah@pnu.edu.sa; 2 Environment and Biomaterial Unit, Health Sciences Research Center, Princess Nourah Bint Abdulrahman University, Riyadh 11671, Saudi Arabia; 3 Department of Biochemistry, Faculty of Science, King Abdulaziz University, Jeddah 21589, Saudi Arabia; fabbasi@kau.edu.sa; 4 Department of Basic Health Sciences, Princess Nourah Bint Abdulrahman University, Riyadh 11671, Saudi Arabia; SJGlani@pnu.edu.sa; 5 Institute of Molecular Biology and Biotechnology, The University of Lahore, Lahore 54000, Pakistan; inamgenetics@gmail.com; 6 Department of Pharmacology, College of Pharmacy, Jouf University, Sakaka 72341, Saudi Arabia; samisz@ju.edu.sa; 7 Department of Pharmacy Practice, College of Pharmacy, AlMaarefa University, Ad Diriyah 13713, Saudi Arabia; mghoneim@mcst.edu.sa; 8 Department of Pharmaceutics, College of Pharmacy, King Saud University, Riyadh 11451, Saudi Arabia; salshehri1@ksu.edu.sa; 9 Department of Biotechnology and Genetic Engineering, Hazara University, Mansehra 21300, Pakistan; geneticsaz@gmail.com; 10 Department of Zoology, Abbottabad University of Science and Technology (AUST), Abbottabad 22310, Pakistan; nazia.murtaza@gmail.com

**Keywords:** aging, life expectancy, genes, genetics, DNA damage repair, signaling pathways

## Abstract

Aging is a complex process indicated by low energy levels, declined physiological activity, stress induced loss of homeostasis leading to the risk of diseases and mortality. Recent developments in medical sciences and an increased availability of nutritional requirements has significantly increased the average human lifespan worldwide. Several environmental and physiological factors contribute to the aging process. However, about 40% human life expectancy is inherited among generations, many lifespan associated genes, genetic mechanisms and pathways have been demonstrated during last decades. In the present review, we have evaluated many human genes and their non-human orthologs established for their role in the regulation of lifespan. The study has included more than fifty genes reported in the literature for their contributions to the longevity of life. Intact genomic DNA is essential for the life activities at the level of cell, tissue, and organ. Nucleic acids are vulnerable to oxidative stress, chemotherapies, and exposure to radiations. Efficient DNA repair mechanisms are essential for the maintenance of genomic integrity, damaged DNA is not replicated and transferred to next generations rather the presence of deleterious DNA initiates signaling cascades leading to the cell cycle arrest or apoptosis. DNA modifications, DNA methylation, histone methylation, histone acetylation and DNA damage can eventually lead towards apoptosis. The importance of calorie restriction therapy in the extension of lifespan has also been discussed. The role of pathways involved in the regulation of lifespan such as DAF-16/FOXO (forkhead box protein O1), TOR and JNK pathways has also been particularized. The study provides an updated account of genetic factors associated with the extended lifespan and their interactive contributory role with cellular pathways.

## 1. Introduction

Aging is a multifaceted, complex process represented by the functional decline of tissues and organs due to changes in molecular composition and physiology of cells. An increase in the human lifespan has been reported for last many decades [1]. According to the estimates of United Nations, there were more than 63 million people above 90 years of age in 2020 [2]. Longevity of human life is heritable from 20% to 40% in the modern human populations [3]. Survival into extremely long ages is a characteristic clustered in families [4]. Better immune systems, metabolic health such as enhanced insulin sensitivity [5], lipid metabolism leading to healthy levels of plasma lipids [6], and delay or ability to avoid age related diseases has also been observed in the longevous families [7,8]. As longevity of life exhibits high heritability, insights into the genetic factors may improve our present understanding of mechanisms responsible to promote health and reduce the risk of diseases [3,9]. However, a few genes and genetic loci have been identified for this trait in the recent studies [10,11,12]. Gene coding for apolipoprotein (APOE) has been reported by multiple genome-wide association studies (GWAS) [10,11,12]. Telomeres are the protective caps at the termini of human and other eukaryotic chromosomes. Their length decreases with age, though it is dependent on many internal and environmental factors. According to reports based on experimental models, the overall length or rate of telomere shortening has been reported to have a link with age expectancy [13]. The shortening of telomere length is rapid in men as compared to women, leading to a comparatively high longevity in women [14]. The application of the well-known antioxidant red wine has been reported to promote the age longevity related genes. Overall, 115 aging associated genes have been studied among 25 mammalian species [15]. Studies on the controlled human populations have shown an increase in the expression levels of Sirt1, catalase, p53 and manganese-superoxide dismutase, after 14 days of red wine use, with no serious side effects. Further studies on *Drosophila melanogaster* have shown up to 7% increase in lifespan after moderate red wine applications [16]. There are several other genes, such as the kinase signaling gene MAP3K5 [17], and variants of genes in the insulin/IGF1 pathway [18]. Studies on bats have shown that DNA methylation is negatively linked with longevity of lifespan, and that DNA damage is positively linked with it [19,20]. Studies on monkeys have shown that the genome flexibility and environmental adaptations also contribute to the lifespan [21]. In context with the above information, we aimed to conduct a systematic review of human genes and their non-human orthologs associated with the life expectancy.

## 2. Genes and Longevity of Lifespan

During past three- or four-decades, genetic researchers have identified many genes that promote lifespan in different species [22,23,24]. Some examples of candidate genes and their orthologs reported for their association with longevity of lifespan have been tabulated in Table 1.

### 2.1. Aging and Genomic Modulation

#### 2.1.1. Apolipoprotein E (APOE)

APOE gene codes for a protein known as apolipoprotein E that binds with the lipid molecules to make lipoproteins. APOE is a pleotropic gene contributing to packing and transport of fats and cholesterol along with human blood, maintenance of normal blood glucose levels in the human body, neuronal cell homeostasis, adrenal and brain physiology and cardiovascular health [229]. There are three extensively studied alleles of the APOE gene, i.e., e2, e3 and e4 coding for Apo E2, E3 and E4 proteins [230,231]. Minor variations of specific amino acids give rise to binding variability in ApoE proteins for multiple corresponding molecules including LDLRs (LDL receptors), HSPGs (cell-surface heparin sulfate proteoglycans), and ABCA1 (ATP-binding cassette protein 1). The amino acid variations also affect protein stability and protein folding [232,233]. About 50% of the world’s population has e3 genotype [234]. APOE is one of the well demonstrated genes that have been clearly linked with human mortality. Studies on European populations have shown up to 3.5% contribution of APOE gene in human lifespan [235]. There are consistent studies demonstrating the role of allele e4 in the onset of Alzheimer disease and cardiovascular health [236,237]. Approximately a 4.2 year shorter lifespan has been found among the populations having high frequency of e4 allele [238]. In a series of experiments Finch, Sapolsky, and Stanford have shown that an interaction between diet and genotype play an important role in the longevity of human lifespan [239,240,241]. They also emphasized that the carriers of e4 alleles have higher cholesterol levels with greater chances of plaque formation in the arteries and enhanced risk of cardiovascular diseases, dementia, stroke and Alzheimer’s disease [230]. There are many factors that contribute to the longevity of lifespan in association with genotypes. Aerobic exercise improves the longevity of human lifespan among e4 allele carriers [242]. The impact of diabetes on the human lifespan is also genotype dependent [243]. There is evidence that the increase in human lifespan started about 1.8 million years ago, at those times the human populations were likely to be homozygous for the e4 allele [244]. Based on several studies, a relationship between cognitive decline and e4 genotype [245], high mortality rate due to pneumonia and severe dementia have been reported in old men with e4 genotype [246]. The e4 type have been reported to have short telomeres that have a role in life expectancy [247]. In Japanese and Italian populations, e2 allele is found to favor and e4 allele decrease the chances of exceptional longevity of lifespan [248]. The main hurdle in the development of ApoE based therapeutics is due to limited understanding of each ApoE (subtype) mediated pathway and its impacts on overall human physiology [25].

#### 2.1.2. p53

p53 is a gene that codes for cell cycle regulatory protein responsible to maintain the genome stability, reduce the mutation rate and suppress cancer. Human tumors have been frequently linked with p53 mutations; about 50% of all tumors involve disruption of this gene [249,250]. The presence of the p53 gene in short lived organisms such as worms and flies, who do not develop cancers, indicate that tumor suppression is not the only function of p53 gene. According to recent studies, p53 and p73 play crucial roles in reproduction [251,252]. There is strong evidence that p53 has the complex role in the regulation of longevity of life in mice, flies, *Caenorhabditis elegans*, and humans [253,254,255], both longevity of lifespan and maturity age for reproduction are always coupled. A short form of p53 also known as p44 has been found associated with the body size and lifespan in mice. Regulated expression of p53 isoforms is responsible to maintain a balance between tissue regeneration and tumor suppression in mammals [256]. DNA damage is considered one of the main reasons behind short lifespan, p53 gene is responsible to maintain the integrity of DNA in species. In the genome of elephants 12 to 20 copies of p53 gene have been reported that are meant to reduce the chances of cancer and DNA damage [257]. (Role of p53 in the regulation of cell division or apoptosis under damaged or mutated conditions is described in the DNA repair section.

#### 2.1.3. Sirtuin (SIRT1)

**Figure 1:**
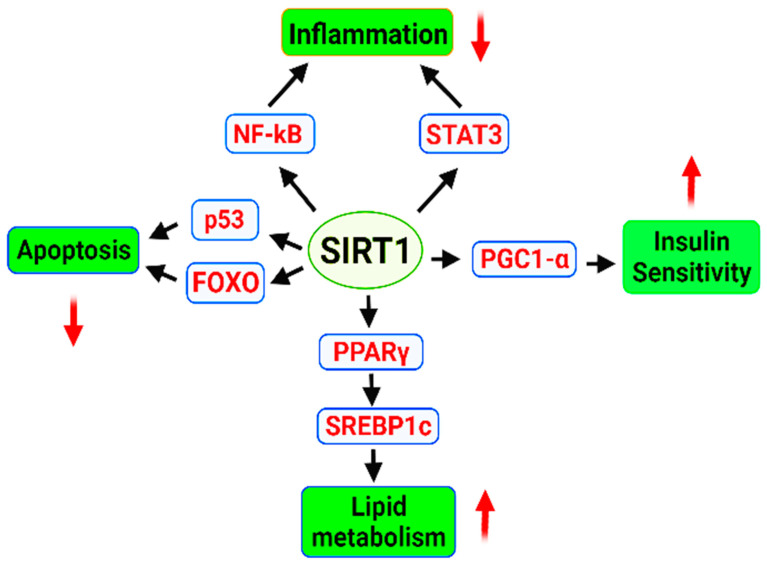
Regulatory role of SRT1 signaling in the cell. SIRT1, an NAD-dependent deacetylase affects several downstream molecules and promotes life. Sirt1 based downregulation of apoptosis and inflammation leads to the extension of lifespan contrary to that enhanced lipid metabolism and insulin sensitivity are main factors that increase the lifespan. As the discussion included the sirt1 gene and we have a separate section for the associated pathways, Figure 1 was meant to indicate the central role of sirt1 gene in the cellular and metabolic pathways involved in the extended lifespan. Green colour indicates final process regulated, downward and upward arrows indicate downregulation and upregulation of process.

Sirtuin (SIRT) is considered an important factor to extend the longevity of lifespan by delaying cellular senescence [258]. SIRT1 is an important member of an NAD+ dependent family of enzymes that have multiple roles in the cells including cell cycle regulation, regulation of energy metabolism, gene silencing and gene regulation [259]. SIRT1 responsible for the deacetylation of tumor suppressor p53 protein, NF-κB and Ku70 (DNA repair factor), is the most well studied isozyme SIRT [260,261,262]. It also regulates FOXO family of transcription factors and STAT3 (signal transducer and activator of transcription 3) [263]. Sirtuin improves the cellular ability to sustain genome integrity by promoting the DNA repair process. It can enhance the ability of a cellular system and makes it resistant the oxidative stress [264]. Increased levels of sirtuin (SIRT2 and SIRT6) have shown prolongevity effect in Drosophila melanogaster, Saccharomyces cerevisiae, Caenorhabditis elegans, and mice [225,265,266]. Overexpression of brain-specific Sirt1 in transgenic mice prolonged their median lifespan by 9% in males and 16% in females [267]. The cellular levels of nicotinamide adenine dinucleotide (NAD+), a “cozymase” can promote DNA repair by reducing metabolic stress and improving mitochondrial function. An increased level of NAD+ in the mammalian and mice cells have shown an activation of SIRT1 and SIRT3 resulting in an increased oxidative metabolism eventually protecting against high fat diet induced problems [268]. The supplementation of precursor molecule of NAD+, NMN (nicotinamide mononucleotide) can reduce the DNA damage, heart failure, and effects of acute renal injury in a SIRT1-dependent manner [269,270]. NMN can help to maintain telomere length, decreases adipogenesis, and improves the osteogenesis in aged mice via SIRT1 activation [271,272]. Administration of NMN has reportedly increased the lifespan in aged mice via (PARP1)/SIRT1 axis [273]. SIRT1 activation reduce insulin resistance, enhances insulin sensitivity via PGC1-α (PPARγ co-activator 1α), implementing beneficial effects in obesity and diabetes type 2 [274,275]. Insulin sensitivity is enhanced by SIRT1 activation via reduction in the expression of proinflammatory genes and it attenuates the insulin resistance induced by tumor necrosis factor alpha (TNF-α) [259,276]. SIRT1 inhibits the production of proinflammatory cytokines via NF-kB [277], and STAT3 [278]. It also exhibits anti-apoptosis activity through p53 and FOXO regulation [279,280,281]. Activation of Sirt1 inhibits lipogenesis and promotes fatty acid β-oxidation [15,282,283] (Figure 1).

## 3. Genomic Instability and Oxidative Stress in Longevity of Lifespan

It has been a universal goal to understand the events and adopt the measures to slow down, stop or reverse the aging process [284]. The most important DNA modification associated with aging is DNA methylation. DNA methylation based biomarkers have answered many of the questions about the aging process and have described the role of epigenetics. As epigenetic changes in the human body are reversible, these biomarkers are therefore useful for the identification and validation of anti-aging interventions [285]. DNA methylation has been linked with human age and the aging process in man and other species. Measurement of changes in methylation (decrease/increase) at a few hundred specific CpG sites can estimate chronological age and mortality [286,287]. Studies have shown that an increased difference between chronological age and DNA methylation age is positively associated with chances of mortality [288,289,290]. Studies by Hannum and Horvath have shown that a difference of 5 years between these two age types can increase the chances of mortality by 11% to 21% [286,287]. An accelerated DNA methylation age significantly increases the risk of can, stroke and cardiovascular diseases [291,292]. The second genetic factor associated with aging is histone modifications. Alterations (decrease/increase) in the methylation of histones have been reported to regulate aging process in *C. elegans* [293]. High levels of trimethylation of H3K4 reduces lifespan of *C. elegans* [294]. Histone methylations have been linked with cellular pathways regulating the age and lifespan. The process of autophagy slows down with age, due to decrease in the ratio of cellular functional mitochondria and subsequent increase in the ROS, aggregation of insoluble or exhausted proteins in the cells. In such cases, avoiding the decrease in autophagy results in the extension of lifespan [295]. Another consequence of the aging process is accumulation of damaged DNA (mutated, irreparable, double stranded breaks) due to decreased efficiency of damaged DNA repair system (DDR system) in the old cells. Histone methylation promotes the DDR of cells and improve the life activity [296]. Acetylation of histones catalyzed mostly by NAD-dependent acetyltransferases is the third important genetic factor associated with the extension of lifespan. Histones are well established role in DNA packaging and regulation of gene expression [297]. Histone modifications are not only associated with rate of transcription but also regulate the precision of transcription process [298]. Acetylated histones are readily available to interact with transcription factors resulting in high levels of transcription [299]. Age associated decrease in histone acetylation is basis of decline in rate of transcription and metabolism leading to shortening of lifespan [300,301].

**Figure 2:**
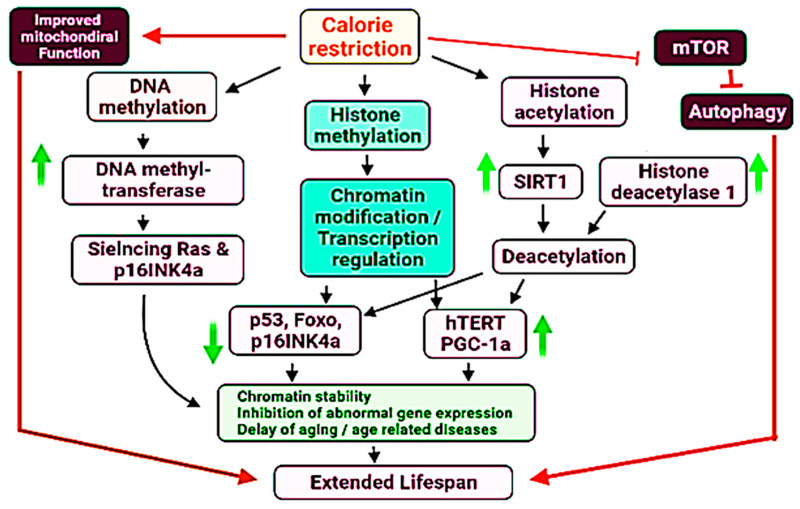
Proposed mechanistic role of DNA methylation, histone methylation and histone acetylation in the regulation of lifespan in the human and other species. Upward and downward arrows indicate upregulation and downregulation of specific genes of processes. Some major aspects have been highlighted by specific colours.

At present, the calorie restriction (CR) and calorie restriction mimetics such as rapamycin have been used as interventions to reduce the difference of methylation based age and chronological age [302]. CR interferes the DNA methylation by activation of DNA methyltransferase, resulting in hypermethylation and silencing of Ras and p16INK4a genes, both of these genes are associated with senescence [303,304]. CR presents an important anti-aging therapy to increase the lifespan of humans, non-human primates and rodents [305,306] (Figure 2).

**Figure 3:**
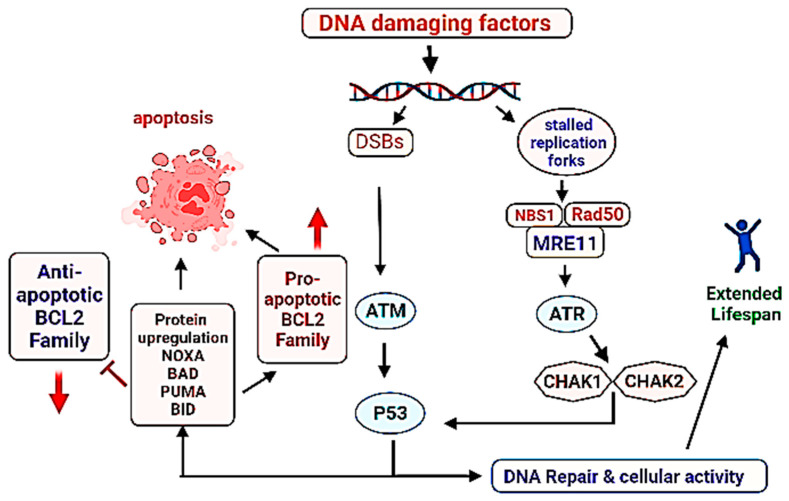
Role of DNA damage repair system in the longevity of lifespan. Successful DNA repair leads to the continuation of life activity/cell division, contrary to that failure in the DNA repair promotes programmed cell death (apoptosis). Direction of arrows indicate upregulation or downregulation of a process.

Another critical factor associated with the survival of a cell is the maintenance of genomic DNA in its intact form. Damaged or mutated DNA accumulation in the multicellular organisms often leads to the onset of cancer or triggers the aging process [307,308,309]. DNA damage triggers aging process by blocking transcription, activating the signal transduction processes, regulating the DNA metabolism, altering the epigenome and finally by inducing apoptosis [310,311,312]. Normally, the DNA damage is recognized by genomic DNA followed by the induction of DNA repair mechanisms such as BER (base excision repair), and double standard breaks (DSBs). If the DNA damage signals continue, the cells select to avoid the replication of mutated or damaged genome rather they prefer to promote the processes leading cell cycle arrest of apoptosis [313,314]. Signaling cascades by the detection of damaged DNA are initiated by the activation of MRN (MRE11/RAD50/NBS1) complex subsequent activation of PIKKs (phosphatidylinositol 3-kinase-like kinases) ATM (ataxia-telangiectasia mutated), ATR (ATM-related kinase) [315,316,317]. ATM and ATR are activated by DNA DSBs and stalled replication forks respectively. Both of these activated factors operate in coordination with 53BP1 MDC1, TOPBP1, and BRCA1 that are sensor proteins responsible to bind the damaged DNA sites and for the recognition of DDR along [318]. TOPBP1 plays a critical role to preserve genome integrity during mitosis [319,320]. After the DNA damage recognition, the transducer kinase check points are phosphorylated and activated such as CHK2 (checkpoint kinase 1) and CHK1 (checkpoint kinase 1), followed by the activation of p53 [321,322] (major roles of p53 in the cellular physiology have already been described in the previous sections). Failure of proper DNA repair mechanism or in case of irreversible DNA damage, the cascades towards programmed cell death are initiated. Activated p53 is recognized as the master regulator of cell cycle, aging, apoptosis. P53 is required for cellular degeneration by genomic DNA damage not required for mitochondrial DNA damage response [323]. In fact a balance of antioxidant and prooxidant activities by p53 in response to oxidative stress play an important role in the longevity of lifespan [324] (Figure 3).

Oxidative stress typically caused by reactive oxygen species (ROS), ultra-violet and ionizing radiations can damage cellular components, especially the lipids, proteins and DNA [325]. Genomic instability includes the events leading to permanent or temporary changes in the genome such as duplications, deletions, inversions and translocations in the chromosomes [326]. Genomic instability may lead to changes in the gene expression, enhance the changes of apoptosis, and it is known as a hall-mark of cancer initiation, age related neurodegenerative diseases [327,328,329]. In the human body, number and diversity of lymphocytes is regulated by the repeated hexanucleotides (TTAGGG) n of 10 kb to 15 kb length known as telomeres [330]. Shortening of telomeres stops the cell division and production of lymphocytes leading to subsequent loss of cellular ability in immune response [331].

Circadian rhythms also play important role in the mammalian cell biochemistry and physiology. Almost each cell has a mechanism to respond to the day night cycles which are mainly regulated by hypothalamus area of brain which is a master pacemaker to modulate cell signaling according to the photoperiods. The circadian desynchrony may lead to the neurodegenerative disorders and metabolic pathologies that ultimately impact the lifespan [332]. The aging process and longevity of lifespan has many well established risk factors including diabetes, obesity, problems of cardiovascular system due to the lack of physical activity. Several epigenetic factors also contribute to the longevity of lifespan [333].

## 4. DAF-16/FOXO (Forkhead Box Protein O1), TOR and JNK Pathways in Aging

### DAF-16/FOXO (Forkhead Box Protein O1), TOR and JNK Pathways

DAF-16 is an orthologous gene coding for FoxO (fork head box transcription factors class O) that regulates the expression levels of genes associated with stress metabolism, aging development and immunity [334,335,336]. There are 19 subclasses of FOX transcription factor in mammals and 6 of them are found in humans [337]. DAF-16 activity is regulated by insulin/ IGF-1 (insulin like-growth factor 1) via IIS (insulin/IGF-1 signaling) pathway [338]. IIS cascade is triggered by the binding of insulin such as peptides (ILPs) to a tyrosine kinase receptor. There are 40 different types of ILPs in *Caenorhabditis elegans* and 10 members of ILPs are found in the human body. IGF-1 and IGF-2 behave similar to those found in *C. elegans* and interact with tyrosine kinase receptor [57,339,340]. These two transcription factors (DAF-16/FOXO and HLH-30) are co-expressed in most of the human tissues particularly in the neuron and intestinal cells [341,342]. For its activity, DAF-16/FOXO directly binds with HLH-30 (helix-loop-helix transcription factor HLH-30) to make a complex to two transcription factors, this binding is conserved in human [343,344]. Activation of DAF-16/FOXO by low IIS triggers the complex formation. DAF-16/FOXO is translocated to the nucleus, this action is mediated by HLH-30 under longevity promoting conditions. Both factors require each other for their action and participate in the coregulation of hundreds of genes associated with the longevity of lifespan [32,345]. The transcription factors DAF-16/FOXO and HLH-30 are not only associated with longevity of life but also play an important role in the stress resistance [346].

**Figure 4:**
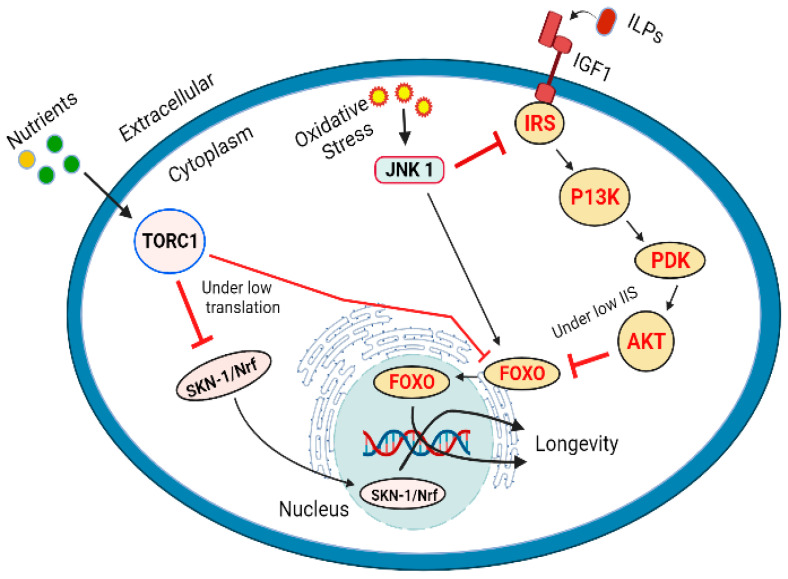
Longevity of lifespan regulated by IIS pathway via DAF-16/FOXO, TOR pathway vis SKN1/Nrf and FOXO regulation, and by JNK under oxidative stress by phosphorylation and inactivation of IRS1 and activation of AKT.

DAF-16/FOXO acts as a joining point for different signaling pathways leading to longevity of lifespan and management of stress. IIS is the basic pathway, highly conserved from *C. elegans*, *Drosophila melanogaster* and mammals [283,347,348]. A signaling cascade initiated by the interaction of insulin such as peptides (ILPs) to the insulin / IGF1 receptor known as (DAF2) results in the downstream activation or regulation of IRS (insulin receptor substrate), P13K (phosphoinositide 3-kinases), PDK (3-phosphoadenosine kinase), and AKT or PKB (protein kinase B), resulting in the inhibition of FOXO. The main consequences of this cascade include the promotion of glucose transport, protein synthesis, cell proliferation, cell differentiation, and inhibition of apoptosis that enhance the longevity. Any stressed conditions or food restrictions leading to the obstruction of IIS signaling pathway will subsequently enhance the DAF-16/FOXO transcriptional activity, regulation of downstream genes resulting to manage stress and increase in the longevity of lifespan (Figure 4).

Target of rapamycin (TOR) kinase which occurs in two distinct forms (TORC1 and TORC2) coded by the same gene. TOR participates to interact between the nutrients and growth promoting (anabolic) metabolic signals [349,350]. TOR pathway is activated by the presence of ample amounts of ATP, oxygen, and amino acids, it triggers the synthesis of nucleotides, lipids, promotes the levels of messenger RNAs and their subsequent translation [351]. TOR signaling is generally responsible for growth and development. However, it has also been linked with aging and diseases such as cancers, cardiovascular diseases, diabetes and neurodegenerative diseases [352,353]. Inhibition of TOR pathway can promote longevity by reducing mRNA translation [354,355,356]. Reduction in the protein synthesis reduces the overall burden on the cellular resources and machinery, it also promotes the expression of genes associated with cell protection. Genetic interference to inhibit the translation process has been linked with the longevity of life in *C. elegans*, the process has been associated with the inhibition of TOR pathway [357,358]. Transcription factor SKN1 (skinhead-1) and DAF-16 genes play an important role in the protection of cells by reducing translation. The similarity in their action suggests the regulation of these transcription factors by TOR pathway [359,360]. Reduced or inhibited TORC1 activity triggers SKN-1/Nrf and DAF-16/FoxO regulated reduction in the stress levels and increased lifespan [360]. The role of SKN-1 is interesting as it is considered as less important transcription factor to DAF-16. IIS inhibits both SKN-1 and DAF-16 by phosphorylation and translocation to the nucleus. However, TORC1 affects the SKN-1 present in the nucleus. A considerable number of SKN-1 molecules are found to occupy specific promoter sites under the conditions without significant stress [361]. Many isoforms of DAF-16 are accumulated in the nucleus when IIS is reduced [36,322,362], but only a single isoform DAF-16f is translocated to the nuclei by the inhibition of TORC1 indicating the most important isoform associated with longevity of life [36]. It has been reported that TORC1 and IIS influence DAF-16/FOXO and SKN1 by different mechanisms. In case of TORC1 induces longevity of life, SKN-1/Nrf are required and presence of DAF-16/FOXO is not obligatory [360] (Figure 4). Under heat stress conditions, JNK-1(Jun N-terminal kinase 1) also indorses the movement of DAF-16 into nucleus, it also facilitates the removal of binding partner 14-3-3 protein by phosphorylation [363,364]. In mammalian cells the FOXO4 can be directly phosphorylated, and its activity can be enhanced by JNK [365]. In mammals, the components of JNK also interact with insulin receptor substrate 1 (IRS-1). According to the reports JNK phosphorylated and inhibited IRS-1 and activated AKT1 [366] (Figure 4).

The well-known signaling pathways based on the NF-κB system that are associated with the human immune system are activated during aging. As the DNA damage in increased by the increased oxidative stress with age, it promotes the activity of NF-κB system that leads to adverse effects such as increase in apoptotic resistance, decrease in autophagy, and an enhanced inflammatory response. A number of inhibitors of NF-κB system are considered as longevity factors [367]. Nuclear receptors are also known to play an important role in the aging process. The NR4A subfamily of orphan nuclear receptors acts as nutrient sensors and promotes biogenesis, improves mitochondrial functioning, and contributes to DNA damage repair. These receptors are considered potential targets to slow down the aging process [368]. Nuclear receptor Nurr1 regulates the development of the dopaminergic phenotype from neuronal precursors. According to recent reports, a decrease in the Nurr1 expression leads to an enhanced inflammatory response resulting in the death of dopaminergic neurons. Hemizygosity of Nurr1 is necessary to protect against Parkinson’s disease [369].

## 5. Conclusions

The lifespan of living organisms is highly flexible and vulnerable to internal physiological factors and environmental conditions that govern the epigenetics. Expression and suppression levels of tens and hundreds of genes have been considered to play a significant contribution to the expected longevity of life. We have tabulated fifty such genes and a few have been elaborated for their influence in the regulation of aging process and lifespan. In addition to the contribution by gene expression regulatory systems, there is sufficient data with diverse evidence to support the critical role of DNA damage in the longevity of lifespan. The integrity of genomic DNA is an obligatory condition to maintain the cellular physiology and continuation of its transfer to the next generations from cell to organism levels. However, DNA is highly vulnerable to the internal and external stress conditions and chemical interferences. DNA modifications such as DNA methylation, acetylation of histones and physical damage to the DNA play important in the aging process and often lead to the unexpected outcomes in the lifespan. The efficient recovery of damaged DNA is crucial to the successful replication and inheritance of genome to the next generations. Failure to the proper DNA damage repair often leads to cell cycle arrest, apoptosis or cell death. The lifespan associated pathways regulate growth, essential physiology and reproduction of organisms. However, under adverse conditions, these pathways shift to protective and stress-bearing modes, eventually leading to an extended lifespan.

## 6. Future Perspective

Currently, research on the contribution of genes to the aging process, cellular stability, and longevity of lifespan is at initial stages. The data available is scattered, and the individual reports provide information about the contribution of selected either a gene or a group of similar genes and genetic mechanisms in the regulation of aging and lifespan, i.e., different reports are available representing the genes responsible for extended lifespan in humans, *C. elegans* and *Drosophila*. Genes regulating the cell cycle, apoptosis, managing the adverse effects of oxidative stress, and their association with life-threatening pathologies such as CVDs, diabetes, and neurodegenerative conditions have been reported but the mechanisms involved mostly remain unclear in the available reports. Large-scale genome-wide association studies (GWAS) in association with epigenetic approaches are required to demonstrate the coordination between different genes, their regulatory factors in the aging process and to reveal the mechanisms. Further studies for the identification of potential genetic targets to protect against aging-associated diseases are also required. Finally, the translation of these genetic findings into clinical practice poses a big challenge.

## Figures and Tables

**Table 1:** Human genes and their orthologues associated with the longevity of lifespan. The main physiological roles of and reported association with life threatening diseases have been discussed for each gene.

Sr. No.	Gene	Protein	Main Physiological Role	Change with Age or Abnormility	Reference
1	APOE	Apolipoprotein E	Odulation of cholesterol balance Aggregability of platelets Proliferation of lymphocytes	Pathogenesis of atherosclerosis and in Alzheimer’s disease	[25,26,27]
2	P53	Tumor protein p53	Tumor suppression Transcription activation	Cancer	[28,29,30]
3	SIRT1	Sirtuin 1 protein	Metabolism and energy homeostasis DNA damage response	Aging, cancer Slow lipid metabolism	[31,32,33,34,35]
4	DAF-16	FOXO1 transcription factor	Transcription factor	Cell cycle arrest, apoptosis	[36,37,38]
5	CHRNA3	Cholinergic receptor nicotinic alpha 3 subunit	Response to foreign materials such as nicotine and alcohol Nervous system	Addiction to alcohol/cocaine Decline in nerve response	[39,40]
6	SH2B3	SH2B adaptor protein 3	Multifunctional adopter protein	Cancer, age dependent Insulin resistance	[41,42,43,44,45]
7	CDKN2A	Cyclin dependent kinase inhibitor 2A	Cell cycle control	Diabetes type 2, obesity, adipose tissue browning, cardiac dystrophy	[46,47,48,49,50,51]
8	ELOVL2	Elongation of very-long-chain fatty acids-like 2	DHA (Docosahexaenoic acid) synthesis, lipid storage	Glucolipotoxicity-induced apoptosis	[52,53,54,55]
9	WRN	Werner protein	DNA protection against oxidative stress damage, genome stability	Premature aging disease, cataracts, atherosclerosis, osteoporosis, and cancer	[56,57,58,59,60]
10	PON1	Paraoxonase 1	Detoxifies Hcy-thiolactone in human blood and reduces chances of atherosclerosis	Cardiovascular disease	[61,62,63,64]
11	SOD2	Superoxide dismutase 2	Fights against oxidative stress ROS scavenger	Alzeimer’s disease, diabetes, cardiac complications	[65,66,67,68]
12	LMNA	Lamin A protein	Regulation of antioxidant associated genes, Impaired protein dysregulation Cardiomyocites function	Accelerated aging process	[69,70,71,72]
13	CETP	Cholesteryl ester transfer protein	Lipoprotein metabolism	Cardiovascular diseases	[73,74,75,76]
14	APOC3	Apolipoprotein C3	Lipid transport Glucose metabolism Neuronal signaling	Insulin resistance Coronary artery calcium and AD	[77,78,79,80]
15	MTP	Microsomal triglyceride transfer protein	Multifunctional protein, cholesterol synthesis, lipd transport lipid and lipoprotein homeostasis	Metabolic diseases	[81,82,83]
16	PIK3CA	Phosphatidylinositol 3-kinase (PI3K).	Neuronal differentiation and survival	Cancer	[84,85,86,87]
17	DAF-2	Insulin-like growth factor 1 (IGF-1) receptor	Anabolic and mitogenic activity	Cancer	[88,89,90,91]
18	PIMT	Protein-L-isoaspartyl methyltransferase	Intrecellular signal transduction	Fatal epilepsy	[92,93,94]
19	GH1	Growth hormone	Growth and development Interaction with insulin-like growth factor	Aging Chronic kidney disease	[95,96,97,98,99,100]
20	KLOTHO	Protein alpha-klotho	Aging suppression Organ protection Phosphate homeostasis Vascular physiology	Chronic kidney and vascular disease,	[101,102,103,104,105,106,107,108]
21	CREB	cAMP-response element binding protein	Neuronal protection, plasticity and memory Triglyceride metabolism	Alzheimer’s disease	[109,110,111,112,113]
22	MAPK	Mitogen-activated protein kinase	Multiple physiological functions Innate immune response Stress signaling	Alzheimer’s disease Parkinson’s disease Cardiac hypertrophy	[114,115,116,117,118,119]
23	EGFR	epidermal growth factor receptor	Nervous system physiology Actin remodeling in sperm capacitation	Cancer	[120,121,122,123,124]
24	NF-kB	Nuclear factor kappa B	Immune response modulator/activator Depression	Coronary artery disease Cancer and immune diseases	[125,126,127,128,129,130]
25	PLC-β	Phospholipase C beta	Signal transduction Brain activity Breast cancer suppression	Neurodegenerative disease; metastasis	[131,132,133,134]
26	MSR-A	Methionine sulfoxide reductase A	Repair of of oxidized methionine in proteins Protects against oxidative stress	Neurodegenerative diseases, cystic fibrosis, neurological disorders, cancer	[135,136,137,138]
27	MEMO1	Mediator of cell motility 1	Cell migration (neuronal cells), Organismal development Central nervous system	Cancer Neurological disorders	[139,140]
28	NEIL1	Nei like DNA Ggycosylase 1	Neurogenesis DNA repair Neuronal protection against oxidative stress	Alzheimer’s disease	[141,142,143]
29	PPARγ2	Peroxisome proliferator-activated receptor gamma 2	Adipogenesis Lipid metabolism	Cardiovascular diseases	[144,145,146,147]
30	EIF3K	Eukaryotic translation initiation factor 3 subunit K	Regulation of apoptosis in *C. elegans*	Lifespan extension in *C. elegans*	[148,149,150]
31	ATM	ATM serine/threonine kinase	Cellular response to genotoxic stress	Lymphoid malignancies Coronary artery disease	[151,152,153,154]
32	BCL2	B-cell lymphoma 2	Multiple functions neuronal activity autophagy calcium homeostasis	Huntington’s disease Chronic obstructive pulmonary disease	[155,156,157]
33	CDC42	Cell division cycle 42	Regulation of mammary gland functioning	Genomic instability Aging	[158,159,160,161,162]
34	DGAT1	Diacylglycerol O -acyltransferase 1	Triglyceride metabolism Promote LDL levels	Congenital diarrheal disorder	[163,164,165,166]
35	EGR1	Early growth response 1	Complex response to stress	Cancer	[167,168,169,170]
36	FGF23	Fibroblast growth factor 23	Phosphate and vitamin D metabolism	Chronic kidney disease	[171,172,173]
37	FGF21	Fibroblast growth factor 21	lipolysis in adipose tissue	Cardiovascular disease	[174,175,176,177,178]
38	FN3KRP	Fructosamine 3 kinase related protein	Cell maintenance and viability Longevity of lifespan	-	[179,180]
39	PGP	Phosphoglycolate phosphatase	Intermediary metabolism	Cardiometabolic diseases	[179,180,181]
40	IRS1	Insulin receptor substrate 1	Insulin signaling	Coronary artery disease Tau pathology in alzheimer’s disease	[182,183,184,185,186]
41	BMI1	Polycomb complex protein BMI-1	Gene silencing Regulation of chromatin structure Cellular bioenergetics DNA damage response	Hematologic malignancies	[187,188,189,190,191]
42	NRG-1	Neuregulin 1	Signaling in the cardiovascular system Maintenance of muscle spindles	Schizophrenia	[192,193,194,195,196]
43	STAT	Signal transducer and activator of transcription	Multiple roles Cell signaling	Autoimmune diseases Cancers	[197,198,199,200,201]
44	E2F1	E2F Transcription Factor 1	Innate immune response Regulation of metabolism	Cancers	[202,203,204,205,206]
45	VEGF-A	Vascular endothelial growth factor A	Cell signale transduction	Cancers	[207,208,209]
46	XME	Xenobiotic metabolizing enzymes	Breakdown of xenobiotic substances	-	[210,211,212,213]
47	MYC	Myc proto-oncogene protein	Maintains regular physiology Tissue repair Placenta development Immune response	Cancer	[214,215,216,217]
48	CXCR4	C-X-C chemokine receptor type 4	Bone marrow physiology	Cancer	[218,219,220]
49	SIR-2	Silent information regulator 2	Extends lifespan in *Drosophila* Human cardiac contractile Suppress neurodegeneration	Neurodegenerative disease Cardiac failure	[221,222,223,224,225]
50	ERK	Extracellular signal-regulated kinase	Cell signaling	Leukemia	[226,227]
51	SLC31		Copper transporters	Immune dysfunction Multiple diseases	[228]
